# The Diverse Pathogenicity of Various *Babesia* Parasite Species That Infect Dogs

**DOI:** 10.3390/pathogens12121437

**Published:** 2023-12-11

**Authors:** Andrew L. Leisewitz, Vladimir Mrljak, Jonathan D. Dear, Adam Birkenheuer

**Affiliations:** 1 Companion Animal Clinical Studies, Faculty of Veterinary Science, University of Pretoria, Pretoria 0110, South Africa; 2 Department of Clinical Sciences, College of Veterinary Medicine, Auburn University, Auburn, AL 36849, USA; 3 Clinic for Internal Diseases, Faculty of Veterinary Medicine, University of Zagreb, 10000 Zagreb, Croatia; 4 Department of Medicine and Epidemiology, School of Veterinary Medicine, University of California, Davis, CA 95616, USA; 5 Department of Clinical Sciences, College of Veterinary Medicine, North Carolina State University, Raleigh, NC 27607, USA

**Keywords:** babesiosis, canine, *Babesia rossi*, *Babesia canis*, *Babesia gibsoni*, *Babesia vogeli*, *Babesia conradae*, disease comparison

## Abstract

*Babesia* species infect a very wide range of mammal hosts across the globe, and zoonotic infections are of growing concern. Several species of the *Babesia* genus infect dogs, and some of these cause significant morbidity and mortality. The Apicomplexan parasite resides within the red cell and infections result in direct damage to the host through intra- and extravascular hemolysis. An exuberant inflammatory response by the host to some species of *Babesia* parasites also results in significant collateral damage to the host. Canine infections have been the subject of many studies as the well-being of these companion animals is increasingly threatened by the spread of tick vectors and an increasingly mobile dog population. There are currently no widely available and effective vaccines, and effective treatment can be challenging. Understanding disease pathogenesis underlies the development of new treatments. The varying pathogenicity of the various *Babesia* parasite species that infect dogs offers an opportunity to explore the molecular basis for the wide range of diseases caused by infection with this parasite genus. In this review, we focus on what has been reported about the clinical presentation of *Babesia*-infected dogs in an attempt to compare the severity of disease caused by different *Babesia* species.

## 1. Introduction

Canine babesiosis is the most common vector-borne infection in many parts of the developing world, where infections are probably very underreported and where veterinary care for dogs is suboptimal. Around 10% of the small animal case load across surveyed practices in South Africa was attributed to canine babesiosis, and that in a country where veterinary care and ectoparasite control are widespread [1]. It is a less common, albeit still important, cause of disease in the developed world. Several species of the *Babesia* genus infect dogs and result in variable clinical manifestations. The literature comparing clinical features of the disease caused by infections with the various *Babesia* species is sparse. Many factors determine the severity of the disease resulting from an infection, and the interplay of these factors is obviously complex. The species of parasite infecting a dog is an important factor and does play a crucial (and probably dominant) role in the nature of the disease seen, and this is the main focus of this review.

It is common for different *Babesia* parasite species to induce clinically distinct diseases. The bovine *Babesia*, *B. bigemina*, causes a relatively mild acute disease followed by persistent infection. *B. bovis*, on the other hand, causes a severe acute disease characterized by neurological signs and a high mortality [2]. The neurovirulence of *B. bovis* is associated with adhesion of infected red cells to the endothelium of the microvasculature of the brain, which initiates inflammation and disturbs normal cerebral blood flow. This is not seen in *B. bigemina* infections.

Classifying a disease severity has typically involved the development of a composite clinical score. Various scoring systems have been developed in both human and veterinary care [3,4,5]. A system has been developed to assess the severity of immune-mediated hemolytic anemia in dogs, a disease with similarities to babesiosis [6]. Scoring systems usually make use of a few crucial and easy-to-measure variables that, through regression analysis, result in the development of a set of numerically weighted criteria that can predict outcomes or assist in assessing disease severity. There is no such scoring system for human malaria or canine babesiosis. Years of research, however, have created awareness amongst clinicians that certain findings are associated with less complicated disease (with a better outcome) or more complicated disease (with a poorer outcome, requiring more intensive treatment). The disease caused by some of the *Babesia* species that infect dogs has been classified as uncomplicated or complicated in a very similar vein to falciparum malaria in humans. Criteria for this classification of canine babesiosis have been published for the disease caused by *Babesia rossi* [7] and *Babesia canis* [8].

The clinical description of babesiosis provided here was gleaned from published case reports and case series of naturally and experimentally infected dogs. Reviews of the literature (i.e., not primary source studies) were seldom used. It is important to recognize that over the last few decades, nomenclature has evolved. Small *Babesia* organisms were morphologically identified as *B. gibsoni* (such as in Conrad et al., 1991 [9]). Molecular techniques now recognize that this report was, in fact, describing *B. conradae* infections [10]. A similar situation arose with the large *Babesia* organisms, which were all typically identified as *B. canis.* An example of this is seen in the work of Malherbe et al. in South Africa, where the very severe babesiosis described was attributed to *B. canis* [11,12,13], which we now know described disease caused by *B. rossi*. More recent molecular techniques recognize three large parasites infecting dogs, namely *B. canis*, *B. vogeli*, and *B. rossi* [14,15]. The early and seminal work by Maegraith et al. published in 1957 [16] attributes the disease they studied to *B. canis* when, in all likelihood, it actually describes an infection caused by a South African isolate now known to be *B. rossi*. It is important to recognize these changes in the identity of the various parasites and, although the work conducted is clearly valid, ascribing it to the correct parasite makes a significant difference to our appreciation of the diseases caused by infection with the various parasite species. *B. vulpes* (previously called *B. annae*, *B. microti*-like, and *Babesia* Spanish isolate [17]) infections in dogs have been reported in the USA and Europe [18,19,20,21,22]. There is some clinical description of the disease (many cases are associated with co-infections) but, where it exists, it is provided. A large *Babesia* parasite that infects dogs (*Babesia* ‘coco’) has been described, but too little is known about this infection to draw conclusions about the clinical manifestation of this infection to compare it to the disease caused by other *Babesia* parasites that infect dogs [23].

The tick vectors for these various canine *Babesia* parasites are provided in Table 1. *B. rossi* is a sub-Saharan parasite [24], *B. canis* is chiefly European, *B. gibsoni* is found in the United States, parts of Europe (especially Eastern Europe), and South East Asia, whilst *B. vogeli* is widely distributed across the world [25]. *B. conradae* has only been described in the USA [26]. *B vulpes* is rarely described and has a spotty distribution in the USA [18], Spain [19], on Prince Edward Island (Canada) [27], and Russia [28].

## 2. Comparing the Clinical Presentations *Babesia* Infections in Dogs Have in Common

Irrespective of which *Babesia* parasite is responsible for an infection, there are features that are broadly similar across the genus. Not all *Babesia* infections make dogs clinically ill. Although probably rare, even in infections with what has traditionally been described as the most pathogenic parasite, *B. rossi* subclinical parasitemia has recently been demonstrated in dogs [29]. The same has been described for *B. canis* [30]. Subclinical infection is common with *B. vogeli* infections [36,37]; however, this is not always true [38]. Asymptomatic *B. canis* infections also seem to be quite common [58]. A significant proportion of *B. gibsoni*-infected dogs are also subclinical [31,32,33,34]. A description of 58 cases of *B. vulpes* infections from Spain describes a severe disease, with no mention of asymptomatic infection [19]. Evidence suggests that for *B. rossi* and *B. canis*, if parasitemia is demonstrated in a sick dog, the default would be to consider the illness to be caused by *Babesia* infection. This is not always true for *B. conradae* [35], *B. gibsoni*, or *B. vogeli* (Table 1).

Lethargy and anorexia are described for infections caused by all species of canine *Babesia*. Vomiting and diarrhea are also occasionally seen. In chronic relapsing infections (such as what is described for *B. gibsoni*), weight loss may be a feature [32,41]. Lethargy and anorexia form part of the owner’s chief complaint in almost all *B. rossi*-infected dogs [7]. It is also described in the majority of *B. canis* infections [8,30,38,40]. These signs are significantly less common in *B. vogeli*-infected dogs [37,38,42]. Lethargy and anorexia are seen in the majority of symptomatic *B. gibsoni*-infected dogs [31]. In a description of 11 naturally infected *B. conradae* dogs, vomiting and lethargy are described as common [9]. *B. vulpes* infection would appear to be associated with obvious illness in a case series of 58 infections and several other case reports [18,19,27,28]. *B. rossi* and *B. canis* infections would appear to be equally likely to cause lethargy and anorexia. *B. gibsoni* may be a subclinical infection, and *B. vogeli* is frequently identified as a subclinical infection (Table 1).

Pyrexia is a very common finding and is typically the result of a host response to an endogenous pyrogen. The association between a fever, TNF production, and the cyclic growth of malaria parasites is a clear example of this [59]. It is described in all cases of *B. rossi* infection, in addition to those which are close to death, where a low rectal temperature is a poor prognostic indicator (probably a terminal shock phenomenon) [7]. In *B. canis*, it is also common but not uniformly present [8,38]. Although *B. vogeli* infection is usually subclinical, pyrexia was common in dogs with evidence of disease [38] and has been shown to resolve despite the persistence of the organism [42]. It is also not a consistent finding in *B. gibsoni* infections, and when it is found, it is described as being poorly correlated with parasitemia [9,32] and seldom rising above 40 °C [41]. There is a poor correlation between the presence of the parasite and pyrexia in infections with this parasite [32,43,44,45,60]. Although pyrexia is noted to occur in *B. conradae* infections, it appears not to be a consistent finding [9,35]. *Babesia rossi* and *B. canis* appear equally likely to induce a fever, whilst in *B. gibsoni*, *B. vogeli*, and *B. conradae*, this is an inconsistent finding (Table 1).

All *Babesia* species that infect dogs can cause anemia. The severity, rate at which anemia develops following infection and the strength of association between infection and anemia do, however, seem to vary. *B. rossi* almost always presents with anemia. Over a third of 320 dogs presented for care were severely anemic (hematocrit < 15%, requiring blood transfusions), a quarter were moderately so, and just under a quarter were mildly anemic. A small proportion of cases had normal hematocrits at presentation. Mortality was only marginally higher in the severely anemic dogs compared to the other groups [7]. All studies that evaluated the hematocrit of *B. canis*-infected dogs described a significantly greater proportion of dogs with mild to moderate anemia, and a very small proportion of these were treated with blood transfusions [8,30,38,47,48]. Case series reporting on the clinical disease caused by *B. vogeli* infections are less common. Anemia is reported but appears to be a feature of the infection in puppies (where it is described as hemolytic and severe) or immunocompromised rather than immunocompetent adult dogs (where the infection is usually subclinical or reported as a co-infection) [38,42,52,53]. *B. gibsoni* causes mild to moderate anemia in the majority of infected dogs [31]. Severe life-threatening anemia is rare [43,44,45,49,50]. There is a more limited description of the clinical disease caused by *B. conradae*, but severe anemia (more pronounced than what is described in *B. gibsoni*) is described [9,50,51]. Moderate regenerative anemia was present in 95% (20/21) cases of *B. vulpes* infections [19]. It would seem from these descriptions that *B. rossi* probably causes a more consistently severe anemia that likely evolves more quickly than what is seen in infections from other parasites. This is followed in severity by *B. canis*, then *B. gibsoni*, *B. conradae*, and *B. vulpes*, with *B. vogeli* being the infection least likely to cause life-threatening anemia.

Together with anemia, hemolysis is described in *B. rossi*. This is, at times, so rapid and overwhelming as to cause black urine (akin to the ‘Black Water Fever’ of falciparum malaria in humans [61]) with port-wine-colored plasma [7,39,62,63]. Cell-free hemoglobin plays a role in disease pathogenesis and is potentially an important measure of disease severity [64]. Eighty-four percent (269/320) of cases had hemoglobinuria at presentation [7]. A rare form of *B. rossi* is associated with hemoconcentration in the face of obvious hemolysis and carries a very poor prognosis [7,39]. Hemolysis is reported to be common in *B. canis* infections with macroscopically visible hemoglobin in urine and/or blood in >2/3 of cases [8], in 24/49 dogs [30], and in 63% of 63 cases [8]. Hemolytic anemia is reported in 11 cases of *B. vogeli* infection, although this is less commonly reported than for either *B. rossi* or *B. canis* [38]. Clinical evidence of rapid intravascular hemolysis is reported for *B. gibsoni* but in a small percentage of cases [49], and evidence for this in *B. vulpes* infections is lacking. It is likely that the more slowly developing infections (such as *B. gibsoni* and *B. vulpes*) result in slower extravascular hemolysis. It appears that *B. rossi* may be responsible for more severe and more sudden hemolysis compared to *B. canis*, although hemolysis in *B. canis* is, nevertheless, clinically obvious in most cases. The other parasites, although reported to cause hemolysis, typically show variable and less marked clinical signs of this process (Table 1).

It is no surprise that dogs infected with *Babesia* develop a reactive splenomegaly, given the spleen’s role as the primary immune organ to detect and remove foreign antigens from the blood. This has been described in *B. rossi* [54], *B. canis* [8], *B. gibsoni* [31], *B. vulpes* [27], and *B. conradae* [35]. Although it has not specifically been described in *B. vogeli* infections, splenectomy has been described to worsen the infection [42]. It is possible that the significantly milder disease caused by this parasite does not evoke the same degree of splenic pathology. A detailed description of splenic pathology has only been reported for *B. rossi* infections [54]. Some cursory comments on the splenic pathology caused by *B. conradae* have also been made and, from these, it appears that the pathology caused by *B. rossi* in the spleen is significantly worse than what is described for *B. conradae* [65].

## 3. Contrasting Features of the Disease Caused by *Babesia* Species

Large case series have been published for both *B. canis* [8,30,48,56,66] and *B. rossi* [7]. These descriptions have allowed for a good understanding of these two infections. Sadly, there is a distinct gap in the description of the gross, histological, and immunohistological pathology of both diseases, although in the case of *B. rossi*, this is slowly being rectified. There are aspects of these two infections that appear to be fairly unique to their clinical presentation and that distinguish them from the diseases caused by the other parasites that need to be highlighted. *B. rossi* is usually referred to as being responsible for the most severe disease. We made use of the markers of disease severity for this infection to compare and contrast with the diseases caused by the other parasites.

### 3.1. Mortality

The greatest majority of *B. rossi*-infected dogs presented for care that die succumb to the infection within the first 24 h of hospitalization, despite intensive treatment [7,39]. Time to death following admission for care has not been reported for other *Babesia* species. From this, it would appear that the rate at which complicated disease develops in *B. rossi* infections is generally much faster than with infections with the other parasites. Mortality in *B. rossi* infections has been reported to range between 5 and 35%, with a rate of over 80% for cerebral or hemoconcentrating cases [1]. Others have reported a mortality of 45% for complicated cases, with death in 10–12% of all admitted cases [55]. Lower mortality rates have also been reported, with 1–3% of cases euthanized because of a grave prognosis and about 5% of all cases dying [39]. In a series of 320 cases, the overall mortality rate was 11%. The odds ratios for death were significantly increased for certain complications (see below). In a large study of *B. canis* infections, 10% of dogs that presented for care were diagnosed with multiple organ dysfunction syndrome, and 67% of these died. Five percent of dogs that did not develop MODS died [56]. The mortality across all 332 dogs included in the study was around 6%—not dissimilar to the *B. rossi* study [7]. Most *B. vogeli*-infected dogs are reported as being subclinical or only very mildly affected [37]. Severe disease (and a single death) were only seen in puppies [38]. Death as a result of *B. gibsoni* infection is rarely reported, and the mortality rate seems very low (not exceeding 5%) [31,32,33,45,49]. In an Indian study, 10% of *B. gibsoni*-infected dogs died, whilst 34% of dual *B. gibsoni/B. vogeli*-infected dogs died. None of the dogs with *B. vogeli* infection alone died [57]. *Babesia conradae* can cause significant mortality, with between 25 and 40% of naturally infected dogs in a study dying or being euthanized because of severe illness in two separate studies [50,51]. Other studies indicate significantly lower mortality than this [9,35], but data from large study populations involving this infection are lacking. From the available mortality data, it is possible that *B. rossi* may more frequently be peracute/acute than *B. canis*, although the mortality rates do not appear dissimilar enough to say with certainty that *B. rossi* is unequivocally more often fatal than *B. canis*. In a case series of 58 *B. vulpes*-infected dogs the mortality rate was very high, with 21/58 (36%) of cases dying within the first week of presenting for care. All of these appear to have succumbed to proteinuric renal failure, which is likely a slowly developing glomerular pathology (Table 1).

An interesting perspective on why *B. rossi* may be so virulent was provided in a recent publication. It is suggested that the domestic dog is a relatively new host to *B. rossi.* The parasite has evolved with the indigenous African black-backed jackal (*Canis mesomelas*), in which it causes no disease. The domestic dog is, thus, a relatively recent ‘spill-over host’ that has had little opportunity to evolve with the parasite [67].

### 3.2. Systemic Inflammatory Response Syndrome (SIRS) and Multiple Organ Dysfunction Syndrome (MODS)

**Figure 1:**
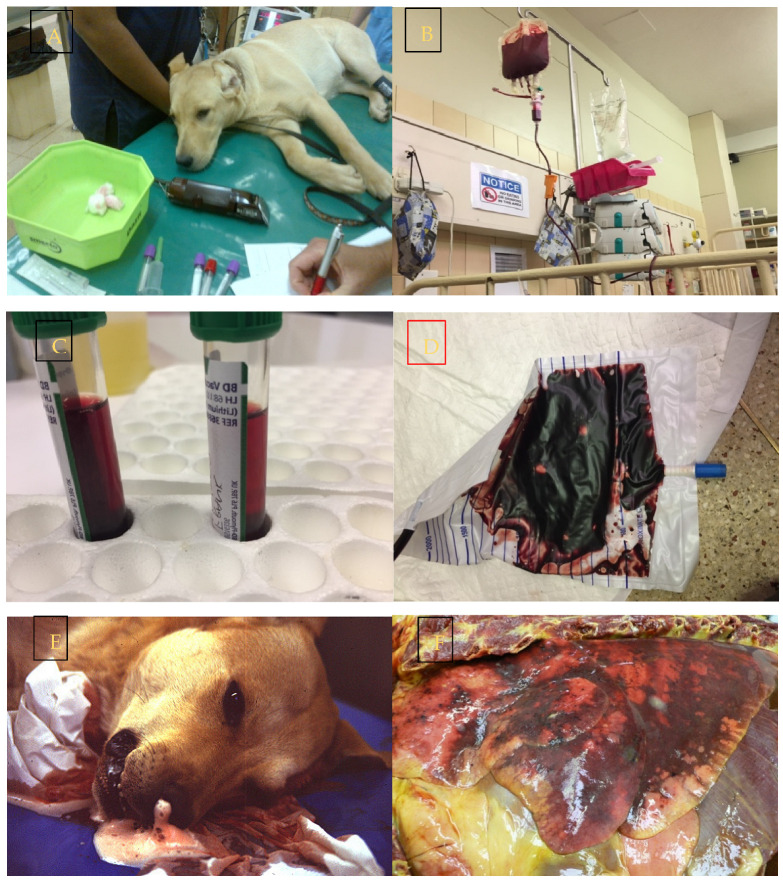

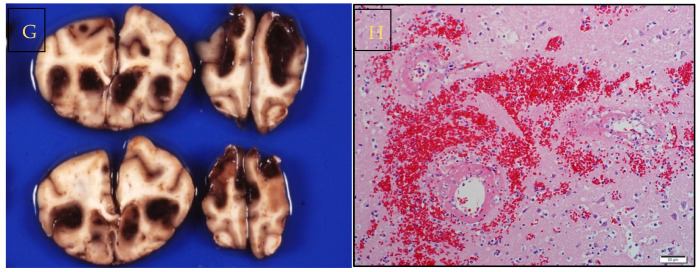
Infection with *Babesia rossi* causes severe disease. (**A**) Collapse at presentation is an indication of severe disease and increases the odds of a poor outcome. (**B**) Blood transfusion is a common form of supportive treatment required in the majority of dogs admitted to a hospital for care. (**C**) Overt signs of intravascular hemolysis are common resulting in hemoglobinemia and (**D**) hemoglobinuria. (**E**) Acute Respiratory Distress Syndrome is associated with a significant risk of death as evidenced here by a blood tinged froth pouring from the nose of an agonal case of *B. rossi* infection. (**F**) On post mortem examination lungs are characterized grossly as edematous with extensive hemorrhage. (**G**) Cerebral babesiosis is uncommon but results in hemorrhagic encephalomalacia. (**H**) Histologically classic ring hemorrhages around small vessels are frequently observed. All images original to the author (AL).

The concept of the Systemic Inflammatory Response Syndrome (SIRS) was developed for use in human medicine in 1991 and was intended to provide physicians with a set of easily measurable clinical parameters that could help identify patients at risk and track host response to a wide variety of insults such as that seen with sepsis [68]. These criteria were adapted for veterinary patients [69]. The usefulness of this syndrome has, however, been questioned [70,71]. Despite this, the veterinary criteria for the identification of SIRS in dogs have been studied in dogs infected with *B. canis* [56,72,73] and *B. rossi* [7,55]. Although this syndrome identifies dogs that are ill with *Babesia*, there are many cases with the syndrome that do not have complicated disease or that succumb to the infection but never qualify to be characterized as having SIRS. A more useful syndrome that identifies multiple organ dysfunction (Multiple Organ Dysfunction Syndrome—MODS) is recognized in human and veterinary medicine [74,75]. Because of the inflammatory and multiorgan nature of babesiosis in dogs, MODS has been studied in *B. canis* [56] and *B. rossi* [7,55]. It should be recognized that just because there is evidence of organ damage (such as elevated liver or muscle enzyme activity), this does not necessarily equate with organ dysfunction. Biochemical evidence of damage does not automatically imply whole organ dysfunction or failure. The more evidence there is of the more organs that are dysfunctional or failing, the more likely a dog is to die of its infection [7,56]. There is also clear evidence for *B. rossi* that certain single organ failures are more predictive of death than a cluster of organs that show biochemical evidence of some level of dysfunction or damage [7,55]. The odds ratio for death (all statistically significant) was 62.39 for cerebral disease, 32.7 for hemoconcentrating disease, 8.36 for a collapsed state at presentation, 4.9 for cases with an increased band cell count, 3.47 for cases that were hypoglycemic at presentation, 7.55 for cases with elevated creatinine, and 2.89 for cases with elevated urea [7]. Neither SIRS nor MODS has been convincingly identified and published for *B. gibsoni*, *B. vogeli*, *B vulpes*, or *B. conradae* infections. Cerebral disease (which carries a very poor prognosis) appears to be more common with *B. rossi* infections. Hemoconcentration, which also carries a very poor prognosis, has only been described in *B. rossi* infections. The fact that single organ failures have such high mortality rates in *B. rossi* infections is likely a reflection of the peracute and severe nature of this infection. The single organ failure (proteinuric renal failure) causing high mortality seen with *B. vulpes* infection is a slowly developing pathology [19] (Table 2; Figure 1).

### 3.3. Organ Pathology

#### 3.3.1. Brain Pathology

In both *B. rossi* and *B. canis* infections, cerebral disease is rare and almost uniformly fatal. The cerebral pathology caused by *B. rossi* has been carefully described [77]. There is a description of the pathology of 56 cases of cerebral babesiosis collected in South Africa over a period of 3 years, presumably caused by *B. rossi*, indicating that this form of *B. rossi* infection may be significantly more common than what is described for *B. canis*. The clinical effect of both infections on the brain appears to be broadly similar, although the incidence of cerebral complications may be more common in *B. rossi* infections. There is also very little reported pathology for *B. canis* cerebral disease for comparison (Table 2; Figure 1).

#### 3.3.2. Renal Pathology

Renal dysfunction appears to be common in both *B. rossi* and *B. canis* infections. Indeed, 91% of *B. canis-*infected dogs identified with MODS demonstrated biochemical evidence of azotemia (elevations in creatinine). It is possible that many of these dogs had a prerenal azotemia, which results in acute kidney injury, albeit potentially more easily remedied than intrinsic injuries [56]. Recoverable renal dysfunction is common in *B. rossi* [63] infections. Elevations in creatinine are not common (just under 10% of 320 cases had elevated creatinine) [7]. This is consistent with the IRIS AKI score of I (http://www.iris-kidney.com/guidelines/grading.html, accessed 30 October 2023). Elevations in creatinine are typically associated with a non-recoverable state of acute renal injury in *B. rossi* infections [7,39] and are typical of an IRIS AKI score of II, III, or IV (http://www.iris-kidney.com/guidelines/grading.html, accessed 30 October 2023). In the early work of Maegraith, anuric renal failure, uremia, and ‘Black Water Fever’ are described in what is now believed to be *B. rossi* and not *B. canis* infection [16]. An elevation in creatinine is a negative prognostic indicator in *B. rossi* infections [7]. Sixteen percent (4/25) of *B. rossi*-infected cases that died had post mortem evidence of nephrosis. Elevations in creatinine are common in severe *B. canis* infection, and this impacts the outcome (in one study, 4/9 dogs with acute renal failure alone died [8]). It would appear that severe glomerular injury (that results in reduced glomerular filtration and increased urea and creatinine concentrations) is a serious complication of *B. rossi* infection, albeit rare. Tubular injury does, however, appear to be common, as shown by Defauw and colleagues [63,106]. Injury is not likely the consequence of cell-free hemoglobin and hemoglobinuria but rather a function of hypoxia and the acute systemic inflammatory response to infection [107]. A reversable protein losing nephropathy has been identified in a small number of *B. gibsoni*-infected American pit bull terriers [81]. An IgM-positive membranoproliferative glomerulonephritis (consistent with a type III hypersensitivity) has been described in *B. conradae* [65]. Increased serum urea concentration without a concomitant increase in creatinine is common in severe *B. rossi* [108]. This suggests that its origins are not reduced glomerular filtration. Increased urea has been correlated with poor outcome [7]. Azotemia with proteinuria appears common in *B. vulpes* infections, with 21/58 (36%) affected in this way. This was associated with 13 of these 21 affected dogs dying within the first week of presenting for care. Azotemia was significantly associated with death [19]. This has not been described for *B. canis* infection. Because the incidence of true acute renal failure is unknown for *B. canis*, it is difficult to say whether, in respect to this organ system, the infections are similar. It is likely that the renal lesion that may result from *B. canis* infection is common but usually reversible, whilst that which results from *B. rossi* infection is less common but more likely to develop into terminal complete renal failure rather than a reversible kidney injury. Acute *B. rossi* infections that rarely result in life-threatening AKI have quite a different pathophysiology to the azotemic and proteinuric renal failure described for the more slowly developing disease seen with *B. gibsoni* and *B. vulpes* infections (Table 2).

#### 3.3.3. Liver Pathology

Although liver dysfunction has been described, this is usually a reflection of modest elevations in various liver enzymes, which are not necessarily a reflection of liver function. Hyperbilirubinemia and icterus are likely to have prehepatic and post-hepatic mechanisms due to the profound hemolytic anemia and hepatosis resulting from infection. The liver was the single most common organ showing biochemical evidence of injury in *B. rossi* [55] and the second most common organ affected in *B. canis* [56] and *B. gibsoni* infections [31,57]. Only 1/11 dogs presenting with *B. vogeli* was icteric [38]. Icterus and hyperbilirubinemia have been reported for *B. conradae;* however, elevated liver enzyme activity appears to be rare [9,35]. The liver pathology in cases of *B. rossi* infection that died was stereotypical and characterized by edema, bile stasis, extramedullary hematopoiesis, vacuolar hepatopathy with some centrilobular necrosis, and mild to moderate macrophage and lymphocytic inflammation [109]. Broadly similar pathology has been described in *B. conradae* [65] infections and may well also be present in *B. canis* infections [8]. Icterus was observed in almost two-thirds of *B. rossi*-infected dogs and elevated serum bilirubin concentrations were predictive of a poor outcome. Icterus was present on post mortem in 68% (17/25) of cases at post mortem and 16% (52/320) of a large cohort study [7]. Icterus is also reported in 80% (39/49) of *B. canis*-infected dogs, although it does not appear to correlate with outcome [30]. Icterus is also common in *B. gibsoni* infections, with the incidence ranging from 14 to 25% of cases [31,44,49]. It is likely that the liver pathology is similar in incidence and severity for *B. rossi*, *B. canis*, and *B. gibsoni* infections and to a lesser degree in *B. conradae* infections. There is no mention of icterus in *B. vulpes* infections (Table 2).

#### 3.3.4. Lung Pathology

Lung pathology that manifests clinically as hypoxemia and is diagnosed clinically as Acute Respiratory Distress Syndrome (ARDS) is rare in babesiosis but has been reported for both *B. rossi* [7,82,83] and *B. canis* [56,110]. There are no references to lung pathology for *B. gibsoni*, *B. vogeli*, or *B. conradae*. Lung pathology in *B. rossi* cases that died is surprisingly common, with acute interstitial pneumonia described in 64% (16/25) of cases on which post mortems were performed. In early work by Maegraith et al. (now believed to be studies of *B. rossi* and not *B. canis*), 74% (25/34) of cases showed lung pathology [16]. The pathology of these lung lesions has also been described, with the lesions being typified by acute interstitial pneumonia characterized by alveolar edema and hemorrhage, with the inflammatory response dominated by mononuclear leukocytes in the alveolar walls and lumens [82]. There are no descriptions of the pathology caused by any other *Babesia* species infection. It seems likely that the lung lesions caused by *B. rossi* are more common and more severe than what has been described for *B. canis*. There is no mention of lung pathology in *B vulpes* infections (Table 2; Figure 1).

#### 3.3.5. Pancreatic Pathology

Pancreatitis is described in a study of *B. rossi*-infected dogs in which 28% of admitted dogs had a canine pancreatic lipase immunoreactivity level (cPLI) in the range diagnosis for pancreatitis [84]. In another study, pancreatitis was histologically confirmed [85]. Pancreatitis was suspected in an old study (based on amylase and lipase concentrations) in 33% of 31 *B. canis*-infected dogs, although it was never the only organ with signs of damage [8]. In 13/46 (28%), an increased lipase DDGR was found, and 8 of these were clinical for pancreatitis (including ultrasound findings) [30]. A much lower incidence (2% of 20 dogs) was described for *B. gibsoni* by means of cPLI activity [86]. There are no reports of pancreatitis in *B. vogeli*, *B. vulpes*, or *B. conradae*. The incidence of pancreatitis appears to be similar for *B. rossi* and *B. canis* but very low or not occurring in infections caused by the other parasites (Table 2).

#### 3.3.6. Coagulopathy

Coagulation and inflammation are inextricably linked and, hence, *Babesia* infections can be expected to result in coagulopathy. Thrombocytopenia (sometimes severe) has been described for all *Babesia* species that infect dogs. Despite this, clinical hemorrhage is very rare, although obvious hemorrhage was common internally during post mortem examination of dogs that died of *B. rossi* infections (seen in 22/25 post mortems [7]). Coagulation has been quite extensively studied in *B. rossi*, in which disseminated intravascular coagulation (DIC) and its association with mortality have been described [87]. Uncomplicated cases of *B. rossi* showed no alteration in coagulation [111]. DIC has also been described in *B. canis* infections [88,89]. There are no descriptions of coagulopathy caused by other species of *Babesia* infecting dogs. It would appear that *B. rossi* and *B. canis* have the ability to cause an equally severe coagulopathy when clotting is evaluated clinicopathologically, but at post mortem, it seems possible that a far more severe coagulopathy is present with *B. rossi* infections (Table 2).

#### 3.3.7. Systemic Inflammation

The very presence of a circulating protozoal infection that results in red cell lysis would dictate that an inflammatory host response would typify a *Babesia* infection. Indeed, much of the pathology seen in babesiosis is arguably caused by an excessive and poorly regulated inflammatory host response; the more severe this host response, the poorer the outcome. Measures of inflammation that are clinically useful include the differential white cell count and the concentration of acute phase proteins, most notably C-reactive protein (CRP). Measuring cytokines is not clinically useful but numerous studies have evaluated these.

The differential white cell count (WCC) has been evaluated in *B. rossi*. Total counts are typically higher in more severely affected dogs but often still within the normal range. Severe leukocytosis is occasionally seen [90,91]. The band cell count, however, was significantly higher in complicated cases and cases that died [7,92]. Interestingly, dogs with complicated disease had significantly depressed CD3^+^, CD3^+^/CD4^+,^ and CD3^+^/CD8^+^ lymphocytes in circulation [93]. This may be consistent with an immune dysregulation consistent with a state of hyperinflammation [112,113]. In *B. canis*, the WCC is usually described as normal or low, with only an occasional finding of an increased band cell count. Lymphopenia has been described but its association with disease severity or immunophenotyping has not been reported [8,30]. In *B. vogeli*, an increase in the band cell count was described in 4/11 cases presenting with illness. An increased WCC was common in the 11 sick dogs [38]. No comment is made about the WCC in other published case series of *B. vogeli* infection. The WCC in *B. gibsoni* infections is generally unremarkable [43,65] or mildly elevated due to neutrophilia, with the dogs more severely affected having moderately elevated counts without remarkable changes in the band cell numbers [31]. There is a report of an obviously inflammatory WCC with an increased band cell count in a *B. gibsoni*-infected dog that was euthanized with ARDS [33]. The WCC in *B. conradae*-infected dogs is described and appears to be varied, though 41% of infected dogs in one study were leukopenic [9,35]. *B. vulpes* also does not seem to be associated with an obvious white cell response [19]. Unfortunately, there are too few cases to draw clear conclusions about the association between inflammation reflected in the WCC and disease severity or outcome. From these data, it would seem that *B. rossi* causes the most severe inflammation, followed by *B. canis*, *B. conradae*, and *B. gibsoni*, which appear comparable, and then, finally, *B. vogeli*, resulting in the least hematologic evidence of inflammation (Table 2).

CRP is a well-established veterinary marker of inflammation in dogs [114]. In *B. rossi*, CRP elevates significantly during infection in concert with disease progression and decreases with resolution but does not predict outcome [92,94]. The levels CRP reaches in *B. canis* infections are similar to what is described for *B. rossi* [30,38,40]. *B. gibsoni* also induces a sudden rise in CRP, which coincides with the appearance of the peripheral parasitemia in experimental infections (which is very delayed compared to similar experimental infections with other parasite species) [43]. In *B. vogeli*, CRP was elevated in 4/5 cases that presented with illness [38]. CRP has not been assessed in *B. conradae* infections. CRP is a sensitive indicator of inflammation and elevates quickly in *Babesia* infections, and it is unlikely to be a good measure to distinguish the disease severity caused by infection with the various *Babesia* parasites (Table 2).

The role of cytokines has been quite widely investigated in the various canid *Babesia* infections. *B. rossi* is an excellent example of a cytokine storm; hyperinflammation and cytokine-mediated immune dysregulation with proinflammatory cytokine levels correlating with disease severity [92,95,96]. Very similar cytokine responses have been described for *B. canis* infections, in which complicated disease and poor outcome are associated with higher concentrations of proinflammatory cytokines [97]. Similar cytokine profiles were described in two experimentally *B. gibsoni*-infected dogs but, as with CRP, the onset of increases coincided with the very delayed onset of parasitemia [43]. There are no published studies that evaluate the role of cytokines in either *B. vogeli* or *B. conradae*. There are only small differences between the cytokine profiles in *B. rossi-* and *B. canis*-infected dogs, making it difficult to decide if cytokine concentrations differentiate the two infections. *B. gibsoni* does, however, appear to drive a less virulent cytokine profile than either *B. rossi* or *B. canis* (Table 2).

#### 3.3.8. Macropathology, Histopathology, and Immunohistochemistry

Evaluation of the gross, histological, and immunohistochemical pathology caused by infection is another way to establish the severity of inflammation and disease. Sadly, despite the incidence of *Babesia* species infecting dogs in many parts of the world, the only infection that has detailed organ pathology described is *B. rossi*. Here, organ pathology is dominated by macrophage/monocyte inflammation, with some tissues showing lymphocyte accumulation. Organ damage has been described in all the organs studied thus far (spleen, bone marrow, brain, liver, and lung [54,77,82,98]). There is a single report describing some pathology caused by *B. conradae* infection [65]. The findings for this infection were similar to *B. rossi* in the liver but changes that appear unique were multifocal segmental necrotizing arteritis seen in the small- and medium-sized arteries of the gastrointestinal tract and muscle [65]. A unique immune complex-mediated glomerulonephritis with proteinuria and azotemia has also been described in 34% of 35 dogs with *B. gibsoni* infection [81]. It seems that the pathology of at least two of these infections has some things in common but some marked differences that reflect a very different pathogenesis. The paucity of basic descriptive pathology of canine *Babesia* infection is an important knowledge gap (Table 2; Figure 1).

### 3.4. Serum Biochemistry Markers of Disease Severity

Other biochemical markers of disease severity include hypoglycemia and hyperlactatemia. Hypoglycemia in *B. rossi* infections is associated with a poor outcome, and hyperglycemia (which is common) is less strongly correlated with a poor outcome [7,99,100]. Hypoglycemia is present in similar proportions of *B. canis*-infected dogs, but its association with outcome is unknown [102]. Hyperglycemia is not described. Hyperlactatemia that is treatment refractory is a good indicator of a poor outcome in *B. rossi* infections [101]. Hyperlactatemic metabolic acidosis is described in *B. canis*, but there is no report on its association with outcome [103]. There is one report of a small number of *B. gibsoni*-infected dogs, in which hyperlactatemia was associated with a poor outcome [57]. Both *B. rossi* and *B. canis* infections can present with mixed acid base disturbances, but *B. rossi* is associated with more severe imbalances than *B. canis* [83,103]. The use of urea, creatinine, and bilirubin was discussed above (Table 2).

### 3.5. Endocrine Markers of Disease Severity

Serum cortisol and thyroxine (T4) are freely available and easily measurable in many small animal clinical settings. Hypercortisolemia and a low T4 in *B. rossi* infections are well correlated with disease severity and have been reported in several studies [7,104,105]. Very similar findings for cortisol have been reported for *B. canis* infections [46]. It is very likely that the endocrine responses described for these two diseases are reflective of the degree of metabolic stress the dogs are enduring. It would be interesting to explore the possibility of T4 and cortisol concentration in *B. gibsoni*, *B. vogeli*, and *B. conradae*. It is possible that these markers of stress would reflect a graded disease severity.

### 3.6. Future Directions

Recognizing that the various canine *Babesia* parasites do, in fact, cause distinct diseases (despite overlap) provides an important basis and justification for exploring the molecular basis for parasite species pathogenicity. It may be possible by comparing the genomes of various *Babesia* parasites with widely varying disease severities that pathogenicity-associated genes or gene families could be identified. This could clarify disease pathogenesis, leading to the identification of mechanisms or gene products amenable to treatment or identifying vaccine targets.

## 4. Conclusions

*B. rossi* appears to cause more severe disease than the other *Babesia* parasites that infect dogs. This is reflected in the speed of disease progression, mortality, nature of organ failure, the rarity of asymptomatic or waxing and waning infections, the severity of the anemia (and likely the rate of fall in hematocrit), and the degree of inflammation (host response) evoked by an infection. *B. canis* appears to be the next most pathogenic parasite, followed by *B. gibsoni*, and, finally, the least pathogenic parasite appears to be *B. vogeli*. Both *B. conradae* and *B. vulpis* appear to be significantly pathogenic, but larger reports describing large case series are needed.

## Figures and Tables

**Table 1:** Comparing the clinical presentations of the disease caused by *Babesia rossi*, *Babesia canis*, *Babesia*, *gibsoni*, *Babesia conradae* and *Babesia vogeli* infection in dogs.

	*Babesia rossi*	*Babesia canis*	*Babesia gibsoni*	*Babesia conradae*	*Babesia vogeli*
**Tick vector**	*Haemophysalis elliptica*	*Dermacentor reticularis*	*Haemaphysalis longicornis* *Haemaphysalis hystricis*	*Ripicephalus sanguineus (?)* *Ornothodoris (?)*	*Ripicephalus sanguineus*
**Assymptomatic illness**	Recently demonstrated [29] but probably unusual.	Has also been shown and may be quite common [30]	A significant proportion are subclinical. In a Korean study, 10% of 60 infected dogs showed no clinical signs [31]. In an experimental infection study, 2/10 dogs remained subclinical after needle infection [32]. In 9 cases described from North Carolina (USA), 1 was parasitemic but healthy [33]. In another American study, 9/10 parasitemic dogs were healthy [34]	Reported in a case series of 29 dogs [35]	Subclinical infection is common; seen in 5/12 cases in one study [36], all 4 cases described in a Chilean study [37], however an Italian study reported 11 dogs with *B. vogeli* infection, and all were ill [38].
**Lethargy, anorexia, vomiting and diarrhea**	Well described in almost all infected dogs [7,39].	Seen in 63/63 cases from Hungary, in 49/49 cases from Germany and 50/50 cases from Croatia [8,30,40]. Anorexia was described in 76% of dogs (23/30) and depression in 93% (28/30) in an Italian study [38] and in 43/50 (86%) cases from Croatia [40]. Anorexia was described in 76% of dogs (23/30) and depression in 93% (28/30) in an Italian study [38].	In the chronic relapsing infections weight loss may be a feature [32,41]. Lethargy was observed in 48/60 (80%) and anorexia in 51/60 (85%) naturally *B. gibsoni* infected dogs from Taiwan [31]. A smaller proportion of these dogs were also noted to have vomiting (18/60) and diarrhea (6/60) as part of their clinical histories [31]	In a description of 11 naturally infected *B. conradae* dogs, vomiting and lethargy are described as common [9]	None of 4 infected dogs from Chile showed any clinical illness [37]. Only splenectomised dogs (*n* = 3) in an experimental *B. vogeli* infection became depressed and anorexic and one of these dogs self-cured [42]. Five of 11 naturally infected dogs from Italy were described as lethargic and anorexic [38]
**Pyrexia**	Well described in almost all infected dogs [7,39].	One study documented pyrexia in 84% (27/32) [8] and another in 43% (13/30) of cases [38].	Pyrexia is also not a consistent feature and is poorly correlated with parasitemia [9,32] and seldom rises above 40 °C [41]. Temperature is frequently described as normal despite parasitemia [32]. In an experimental infection pyrexia developed on days 13 and 14 post infection before parasites were seen on blood smear. Fever also resolved within days and never recurred despite a climbing parasitemia [43]. In another study, 8/10 dogs developed a transient fever which mostly resolved [32]. A study of 79 naturally infected dogs in India did not regard it as a cardinal clinical finding as only 31% (5/16) of dogs with positive blood smears were febrile [44]. In another study pyrexia was only detected in 2/8 naturally infected dogs that were anemic and PCR positive for *B. gibsoni* DNA [45].	Pyrexia is noted to occur in *B. conradae* infections, it appears not to be a consistent finding [9,35]	Pyrexia was common in 11 dogs with evidence of disease [38]. All 8 dogs experimentally infected showed a mild pyrexia which self-resolved in a matter of days despite the infection persisting [42]. Five of 11 naturally infected Italian dogs developed pyrexia [38]. Five of 11 naturally infected Italian dogs developed pyrexia [38]
**Anemia**	Over a third of 320 dogs were severely anemic (hematocrit < 15%, requiring blood transfusions), a quarter were moderately so, and just under a quarter were mildly anemic. A small proportion of cases had normal hematocrits at presentation [7]	A significantly proportion of dogs reported with mild to moderate anemia and a very small proportion of these were treated with blood transfusions [8,30,38,46,47,48].	Caused anemia in over 80% of 60 infected dogs. A quarter of these dogs had mild to moderate anemia whilst just over 10% of dogs had hematocrits < 20%. Similar findings in other studies describe a severe life-threatening anemia rarely with mild to moderate anemia being more characteristic of the infection [43,44,45,49,50].	Severe anemia is described in 11 cases (before *B. gibsoni* and *B. conradae* were understood to be separate species) [9]. The anemia is described as more pronounced than what is observed in *B. gibsoni* infections [50]. One study from California demonstrated mild anemia in 13/29 infected dogs [35] while another demonstrated severe anemia in 3/12 infected dogs [51].	Anemia is reported but appears to be a feature of the infection in puppies (where it described as hemolytic and severe) or in immunocompromised rather than immunocompetent adult dogs (where the infections is usually subclinical or reported as a co-infection) [38,42,52,53].
**Hemolysis**	84% (269/320) of cases had hemoglobinuria (as a result of massive intravascular hemolysis) at presentation [7]	Reported to be common with macroscopically visible hemoglobin in urine and/or blood in >2/3rds of cases [8], in 24/49 dogs [30] and in 63% of 63 cases [8]	Evidence of hemolysis is reported but in a small percentage of cases [49]	Not reported/unknown	Hemolysis is reported in 11 cases [38]
**Splenomegaly**	Well described [54]. A detailed description of splenic pathology has been reported [54]	Well described [8]	Well described [31]	Has been described [35]	Not specifically described.
**Mortality**	The majority of infected dogs that die, succumb within the first 24 h of hospitalization, despite intensive treatment [7,39]. Ranges between 5 and 35% with a rate of over 80% for cerebral or hemoconcentrating cases [1]. Others have reported mortality of 45% for complicated cases with death in 10–12% of all admitted cases [55]. Lower mortality rates have also been reported with 1–3% of cases euthanized because of a grave prognosis and about 5% of all cases dying [39]. In a series of 320 cases the overall mortality rate was 11%	In one study 10% of dogs were diagnosed with multiple organ dysfunction syndrome and 67% of these died. Five percent of dogs that did not develop MODS died [56]. The mortality across all 332 dogs included in the study was around 6%.	In a Korean study of 9/39 dogs (31%) were regarded as subclinical and none were reported to have died [49]. In a study of 60 infected dogs from Taiwan, the majority of dogs had mild to moderate disease, 10 dogs were severely anemic and relieved a blood transfusion and no deaths were reported [31]. In an American study of 150 cases, most were reported as mild or moderate disease and there were no reported deaths [45]. One of 9 naturally infected dogs from North Carolina (USA) died [33]. In an Indian study, 10% of *B. gibsoni* infected dogs died whilst 34% of dual *B. gibsoni/B. vogeli* infected dogs died. None of the dogs with *B. vogeli* infection alone died [57]. In an experimental infection study, 2 of 9 infected dogs died [32]	Can cause significant mortality with between 25 and 40% of naturally infected dogs dying or being euthanized because of severe illness in two separate studies [50,51]. Other studies indicate a significantly lower mortality than this [9,35] but data from large study populations involving this infection are lacking.	Most cases reported as subclinical or only mildly affected [37]. No dogs in an experimental infection died [57]. One of 11 dogs in a case series died [38]. Severe disease (and the single death) were only seen in puppies [38].

**Table 2:** Markers of inflammation and organ pathology. (SIRS: Systemic Inflammatory Response Syndrome; MODS: Multiple Organ Dysfunction Syndrome).

	*Babesia rossi*	*Babesia canis*	*Babesia gibsoni*	*Babesia conradae*	*Babesia vogeli*
**SIRS and MODS**	SIRS has been described [7,55] MODS has been described [7,55]	SIRS has been described [56,72,76] MODS has been described [56]	Neither SIRS or MODS have been described	Neither SIRS or MODS have been described	Neither SIRS or MODS have been described
**Brain pathology**	Cerebral disease well described [77]	Cerebral disease described [78,79].	Not described	Not described	Not described
**Renal pathology**	Recoverable renal injury is common [63]. Severe irreversible renal failure is uncommon but occurs and carries a very poor prognosis [7,16,39]	Renal injury is described [56,80]. In one study 4/9 dogs with acute renal failure alone died [8]	A reversable protein losing nephropathy has been identified in a small number of infected American pit bull terriers [81]	An IgM positive membranoproliferative glomerulonephritis (consistent with a type III hypersensitivity) has been described [65]	Not described
**Liver pathology**	The liver was the single most common organ showing biochemical evidence of injury [55]. Icterus was observed in almost two thirds of infected dogs and elevated serum bilirubin concentrations were predictive of a poor outcome. Icterus was present on post mortem in 68% (17/25) of cases and 16% (52/320) of a large cohort study [7]. Acute lung injury (ALI) is common with all dogs that died in one study demonstrating it [82]	The liver the second most common organ showing biochemical evidence of injury [56]. Icterus is also reported in 80% (39/49) of infected dogs although this does not appear to correlate with outcome [30]	Evidence of liver injury is described [31,57]. Icterus is common in with the incidence ranging from 14–25% of cases [31]	Icterus and hyperbilirubinemia has been reported however elevated liver enzyme activity appears to be rare [9,35].	Only 1/11 dogs presenting ill was icteric [38].
**Lung pathology**	Acute lung injury (ALI) is common with all dogs that died in one study demonstrating it [82] Acute Respiratory Distress Syndrome (ARDS) is rare but has been reported [82]. The proportion of dogs with ARDS was 18% (18/98) of cases having an arterial pO_2_ < 60 mmHg in one study [7] and 9% (3/34) in another [83].	Acute Respiratory Distress Syndrome (ARDS) is rare but has been reported [82]. 16/331 (just <5%) of dogs demonstrated diagnostic criteria consistent with ARDS [56]	Not reported	Not reported	Not reported
**Pancreatic pathology**	Diagnosed in 28% of admitted dogs based on pancreatic lipase immunoreactivity level (cPLI) [84]. In another study, pancreatitis was histologically confirmed [85]	Suspected in an old study (based on amylase and lipase concentrations) in 33% of 31 infected dogs although it was never the only organ with signs of damage [8]. In 13/46 (28%) an increased lipase DGGR was found and 8 of these were clinical for pancreatitis (including ultrasound findings) [30].	Described in 2% of 20 dogs by means of canine specific pancreatic lipase activity [86]	Not reported	Not reported
**Coagulopthy**	Hemorrhage was common internally during post mortem examination of dogs that died (seen in 22/25 post mortems [7]. Disseminated intravascular coagulation (DIC) and its association with mortality has been described [87].	DIC has been described [88,89].	No description	No description	No description
**White cell count**	Total counts are higher in the more severely affected dogs but often still within the normal range. Severe leukocytosis is occasionally seen [90,91]. The band cell count was significantly higher in complicated cases and cases that died [7,92]. Complicated disease had significantly depressed CD3^+^, CD3^+^/CD4^+^ and CD3^+^/CD8^+^ lymphocytes in circulation [93].	WCC described as normal or low with only an occasional finding of an increased band cell count. A lymphopenia has been described but its association with disease severity or immunophenotyping have not been reported [8,30].	The WCC is generally unremarkable [43,65] or mildly elevated due to neutrophilia with the dogs more severely affected having moderately elevated counts without remarkable changes in the band cell numbers [31].	The WCC in infected dogs appears to be varied though 41% of infected dogs in one study were leukopenic [9,35].	An increase in the band cell count was described in 4/11 cases presented with illness. An increased WCC was common in the 11 sick dogs [38].
**C-reactive protein**	Elevates significantly during infection in concert with disease progression and decreases with resolution, but does not predict outcome [92,94]	Elevates significantly with infection and decreases with resolution but does not predict outcome [30,38,40].	Infection induces a sudden rise which coincides with the appearance of the peripheral parasitemia in experimental infections (which is very delayed compared to similar experimental infections with other parasite species) [43].	Unknown	Elevated in 4/5 cases that presented ill [38]
**Cytokines**	Induces a cytokine storm, hyperinflammation and cytokine mediated immune dysregulation with proinflammatory cytokine levels correlating with disease severity [92,95,96]	Induces a cytokine storm in which complicated disease and poor outcome are associated with higher concentrations of proinflammatory cytokines [97]	Cytokine profiles were described in 2 experimentally *B. gibsoni* infected dogs but, as with CRP, the onset of increases coincided with the very delayed onset of parasitemia [43].	Not described	Not described
**Macropathology, histopathology and immunohistochemistry**	Organ damage has been described in all the organs studied thus far (spleen, bone marrow, brain, liver and lung [54,77,82,98]).	No descriptions	A unique an immune complex mediated glomerulonephritis with proteinuria and azotemia has also been described in 34% of 35 dogs with *B. gibsoni* infection [81]	A single report describing some pathology caused by *B. conradae* infection [65]	No descriptions
**Blood glucose and lactate**	Hypoglycemia (present in around 23% of complicated cases) is associated with a poor outcome and hyperglycemia (which is common) is less strongly correlated with a poor outcome [7,99,100]. Hyperlactatemia that is treatment refractory is a good indicator of a poor outcome [101].	Hypoglycemia is present in around 20% of complicated cases but its association with outcome is unknown [102]. Hyperlactatemic metabolic acidosis is described but there is no report on its association with outcome [103].	There is one report of a small number dogs in which hyperlactatemia was associated with a poor outcome [57].	Nor reported	Not reported
**Endocrine markers of disease severity**	Hypercortisolemia and a low thyroid hormone are well correlated with disease severity and have been reported in several studies [7,104,105]	Hypercortisolemia and a low thyroid hormone are correlated with disease severity and have been reported [46].	Not reported	Not reported	Not reported

## Data Availability

No new data were created or analyzed in this study. Data sharing is not applicable to this article.
